# Nanoscale C–H/C–D mapping of organic materials using electron spectroscopy

**DOI:** 10.1038/s41565-025-01893-5

**Published:** 2025-03-24

**Authors:** Ryosuke Senga, Katsumi Hagita, Tomohiro Miyata, Hsiao-Fang Wang, Koichi Mayumi, Hiroshi Jinnai, Kazu Suenaga

**Affiliations:** 1 Nanomaterials Research Institute, National Institute of Advanced Industrial Science and Technology (AIST), Tsukuba, Japan; 2 SANKEN (The Institute of Scientific and Industrial Research), The University of Osaka, Ibaraki, Japan; 3 Department of Applied Physics, National Defense Academy, Yokosuka, Japan; 4 Institute of Multidisciplinary Research for Advanced Materials, Tohoku University, Sendai, Japan; 5 Department of Chemical and Materials Engineering, National Central University, Taoyuan City, Taiwan; 6 The Institute for Solid State Physics, The University of Tokyo, Kashiwa, Japan

**Keywords:** Polymer characterization, Transmission electron microscopy, Characterization and analytical techniques, Molecular dynamics

## Abstract

Distinguishing hydrogen from deuterium using atomic-scale imaging and spectroscopy is crucial for identifying microscopic structures and the origins of the properties of organic materials. However, conventional structural analysis techniques for materials containing both isotopes, such as neutron scattering, provide only averaged information across the beam area. Here we utilize vibrational spectroscopy using a monochromated transmission electron microscope to discretely image hydrogen and deuterium in organic polymers at single-nanometre resolution. This technique allowed carbon–hydrogen and carbon–deuterium stretches to be mapped, which uncovered the surface segregation of the deuterated polystyrene component within a block copolymer film composed of deuterated polystyrene and poly(2-vinylpyridine). Moreover, it enabled clear visualization of the spatial distribution of hydrogenated and deuterated polystyrene on a molecular scale in a bulk block copolymer specimen containing both components. This method, integrated with coarse-grained molecular dynamics simulations, revealed a localized feature of polymer chains corresponding to the reptation tube, which could not be identified using conventional scattering techniques.

## Abstract (web summary)

Carbon–hydrogen and carbon–deuterium bonding in organic polymers were mapped in real space with single-nanometre spatial resolution using a monochromated transmission electron microscope.

## Main

Deuterium (D), a stable isotope of hydrogen with one proton and one neutron, was discovered in 1932^1^. It has been extensively used as an isotopic label while performing structural analyses of organic materials by neutron scattering, mass spectrometry and nuclear magnetic resonance. Site-specific deuteration is particularly useful for elucidating structures of multicomponent protein complexes^2,3^ and is applied in pharmacology to track metabolic processes^4,5^. In polymer science, deuterated labelling aids in studying polymer chain conformations, polymer–polymer miscibility and dynamics in melt states via neutron scattering^6–10^. In addition, the unique physical properties of deuterated compounds, such as distinctive vibrational spectra and stronger binding energies, have been leveraged in designing functional materials for plastic optical fibres^11,12^ and neutron sources^13–15^.

Small-angle neutron scattering (SANS) is extensively used to provide statistical structural information for organic materials, spanning from a few nanometres to several hundred nanometres in reciprocal space^6–10^. SANS distinctly identifies deuterated organic molecules from their hydrogenated ones in terms of their differing neutron scattering cross-sections, despite their nearly identical chemical properties^6–10^. This contrasts with X-ray and electron scattering techniques, in which the scattering cross-sections are largely unaffected by the neutron count. However, SANS yields only averaged information in real space and fails to reveal local nanostructures owing to the millimetre-scale width of the neutron beam. Molecular dynamics (MD) simulations are often used to model real-space nanostructures that align with the scattering data^16^. However, validating these models requires them to be compared with actual local structures observed in real space. This means that real-space deuterium/hydrogen (D/H) imaging as a counterpart to SANS is necessitated for establishing a bidirectional correspondence between localized and averaged structures.

Transmission electron microscopy (TEM), particularly annular bright-field scanning TEM (ABF-STEM), can be used to capture real-space images of light elements, including hydrogen^17^. However, its application in hydrogen imaging is confined to well-oriented crystals. Furthermore, TEM techniques, including ABF-STEM, rely on electron scattering and diffraction and cannot inherently differentiate between isotopes. In addition, probe-based spectroscopic methods, such as near-field Fourier-transform infrared (FTIR) spectroscopy, can be used to detect light atoms and isotopes at the nanometre scale based on localized molecular vibrational spectra^18^, but these techniques primarily provide surface-level information.

The advent of transmission electron microscopes with monochromators has enhanced energy resolution, enabling the direct capture of vibrational absorption spectra from both organic and inorganic substances by electron energy loss spectroscopy (EELS)^19–22^. The application of EELS, especially with aloof geometry, to organic materials like biomolecules has been important because it minimizes sample damage by preventing direct electron beam contact^23–25^. This approach allows the molecular vibrations of chemical bonds involving hydrogen to be distinguished from those with deuterium, based on vibrational energy differences^26–28^. However, the vibrational spectra obtained through bright-field (on-axis) EELS, even in aloof mode, often suffer from extended signal delocalization over several hundred nanometres. Consequently, a strong demand exists for truly localized mapping of hydrogen and deuterium using dark-field EELS (DF-EELS), which offers atomically localized vibrational spectroscopy^29–33^.

In our study, we discretely imaged hydrogen and deuterium in organic polymers at the single-nanometre scale using DF-EELS using a monochromated transmission electron microscope. The mapping of vibrational spectra for carbon–hydrogen (C–H) and carbon–deuterium (C–D) stretches enabled deuterated compounds to be distinctly differentiated from hydrogenated ones in block copolymers. Furthermore, by integrating real-space C–D/C–H imaging with DF-EELS and coarse-grained (CG) MD simulations, we accomplished molecular-level characterization of organic polymers. Our approach uncovered the surface segregation of deuterated polystyrene (dPS) in a dPS-*b*-poly(2-vinylpyridine) (P2VP) block copolymer (BCP), attributed to slight differences in the surface free energy. Such wraparound behaviour has not been observed using other imaging or scattering techniques, including SANS. In addition, we observed the real-space structure of a quenched melt of the dPS-*b*-hPS BCP, at resolution nearing the rheologically characteristic size defined by the reptation theory, as is indicative of the diffusional motion of polymer chains^34,35^. Detection by deuterium labelling at such high spatial resolution enables the visualization of the spatial distribution of individual molecules and chemical bonds in polymers. This provides valuable insights into the origins of the functionalities of biomolecules and widely used polymeric materials, including crosslinked rubbers and epoxy resin adhesives, which were previously understood only with the aid of macroscopic characterizations.

## Spectral imaging of dPS-*b*-P2VP

**Fig. 1:**
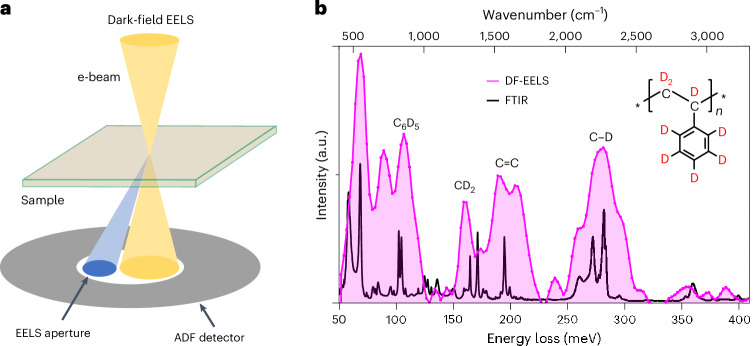
DF-EELS of dPS. **a**, Electron beam geometry of DF-EELS. **b**, Vibrational spectrum of dPS thin film collected by DF-EELS (purple) compared with the FTIR spectrum (black) of the same sample. The chemical structure of dPS appears as an inset in **b**. The EEL spectrum from which the background has been subtracted is presented in Extended Data Fig. 1. Source data

**Extended Data Fig. 1:**
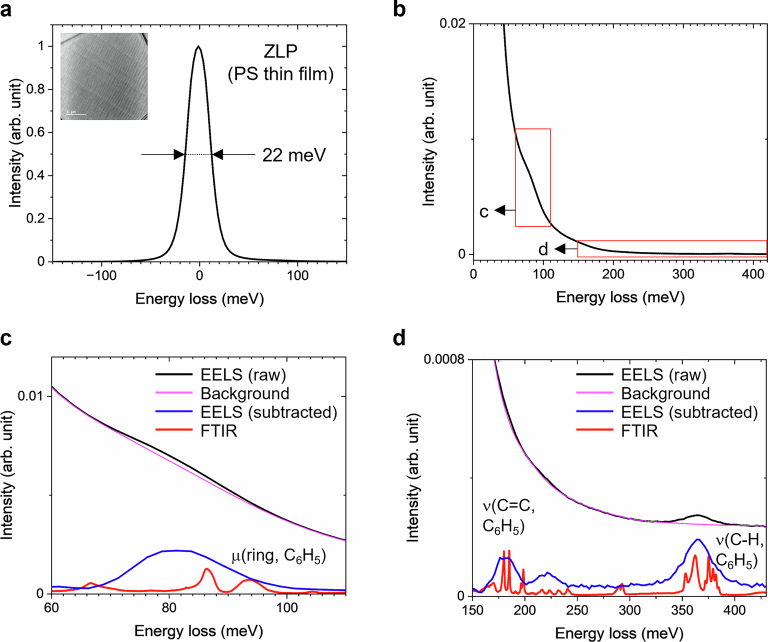
Bright-field vibrational spectroscopy of hPS compared to FTIR spectroscopy. **a**,**b**, Quasi-elastic scattering peak and energy loss in the IR region obtained from the hPS thin film via BF-EELS. **c**,**d**, Enlargements of the spectral regions demarcated in red in (**b**). The signal spectrum (blue curve) is obtained by extracting the background (pink curve) from the raw spectrum (black curve). The power-law background model is applicable to energy loss higher than 300 meV. However, energy loss lower than 300 meV is barely extracted by a single power-law function, owing to the tail of the quasi-elastic scattering peak. The signal below 300 meV is derived by extracting the background interpolated using a spline curve. Source data

Vibrational spectroscopy under off-axis DF conditions is governed by impact scattering and offers highly localized information (Fig. 1a). The spatial resolution of off-axis DF-EELS is ~0.3 nm (ref. ^33^). Figure 1b compares the vibrational spectrum of dPS specimens obtained by DF-EELS with the FTIR spectrum. All EELS measurements in this study were performed at −120 °C to compromise both electron beam damage and ice contamination. The major peaks corresponding to C–D (CD, CD_2_ and C_6_D_5_) and C=C stretching in both the EELS and FTIR spectra were consistent in terms of their bond energies. However, their fine structures differed slightly, which cannot be explained only by the gap in energy resolution. This is primarily because DF-EELS uses different selection rules that are distinct from those of bright-field EELS (BF-EELS) and activate additional vibrational modes that are inactive in the long-wavelength limit (Extended Data Fig. 1). Notably, the selection rules for EELS and FTIR are not identical, even under BF-EELS conditions, owing to the non-zero momentum transfer by the incident electrons. In fact, subtle differences between the spectra obtained by these two methods have been observed in previous studies^23–28^.

**Fig. 2:**
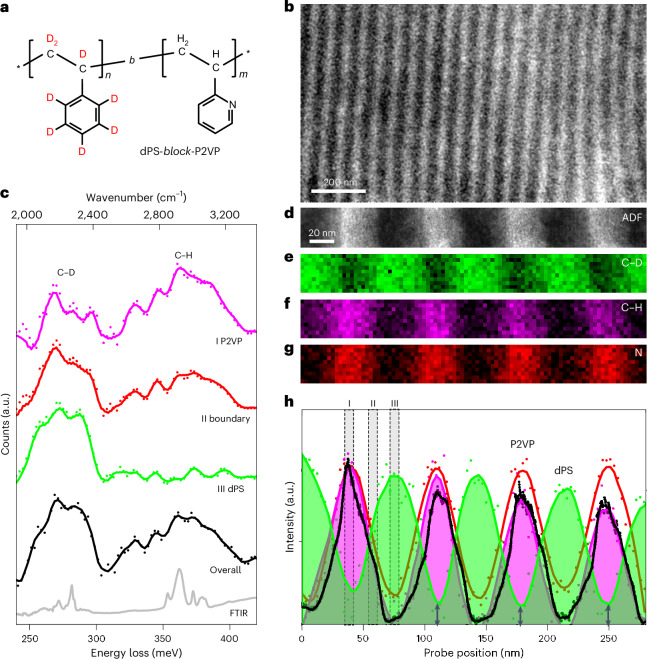
Vibrational spectroscopy of isotopically labelled block copolymer. **a**, Chemical structure of the block copolymer comprising dPS and poly(2-vinylpyridine) (dPS-*b*-P2VP). **b**,**d**, STEM-ADF images of the lamellar structure of dPS-*b*-P2VP at low (**b**) and high (**d**) magnifications. **c**, Vibrational spectra of C–D and C–H stretches obtained from the P2VP domain (purple), dPS domain (green), its boundary (red) and the whole region in **d** (black) as well as the FTIR spectrum (grey). **e**,**f**, C–D (**e**) and C–H (**f**) maps corresponding to the images shown in **d** (31 nm × 281 nm rectangular region). The area in **d** was scanned using a grid of 10 × 91 pixels (3.1 nm per pixel), with each parent pixel further subdivided into a 16 × 16 sub-scan. **g**, Nitrogen map derived from the N K-edge (Extended Data Fig. 2d) taken from the same area. **h**, Intensity profiles of ADF, C–D, C–H and N corresponding to the maps shown in **d**–**g**. The Roman numbers (I–III) assigned to the spectra in **c** correspond to the labels of the integrated regions in **h**. The spectral images were processed by the principal component analysis algorithm using five components. The background subtraction and signal intensity from single-pixel analysis are presented in Extended Data Fig. 4. Source data

**Extended Data Fig. 2:**
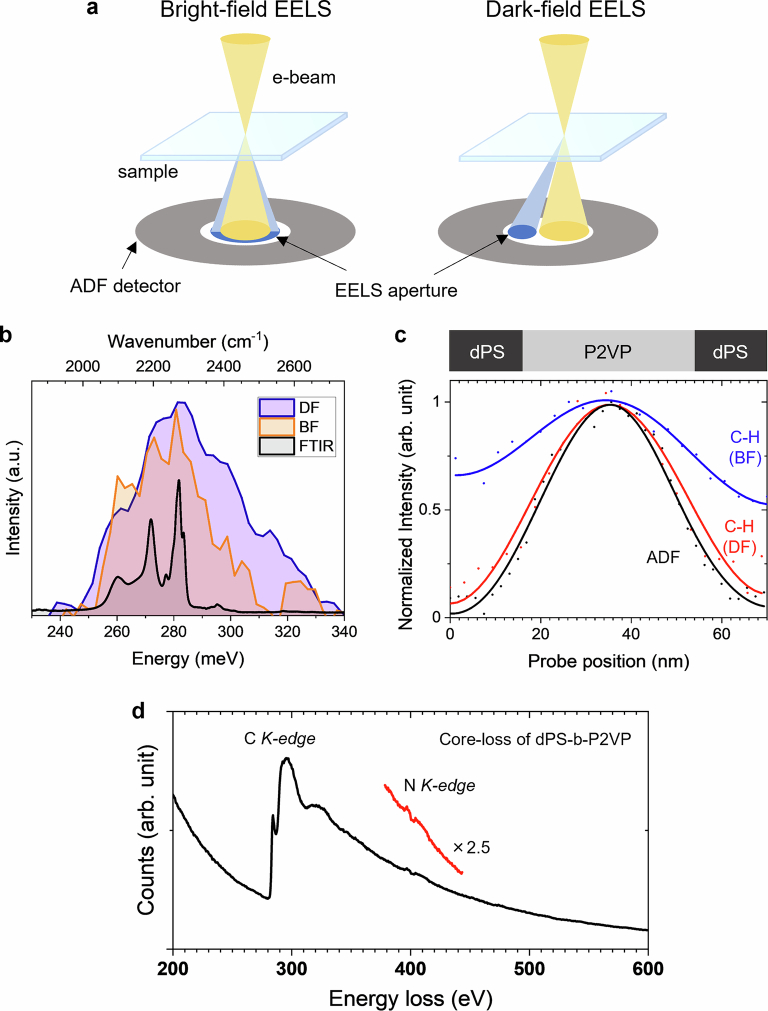
Comparison between BF- and DF-EELS for molecular vibrational spectroscopy. **a**, Electron beam geometry of BF- (left) and DF-EELS (right). **b**, Vibrational spectra of the C–D stretches in dPS. BF- (orange) and DF- (purple) EEL spectra compared with the FTIR spectrum (black) of the same specimen. Because the specimen was not crystalline, the vibrational modes had no dispersion relationship. Therefore, the DF-EELS may reflect the optically forbidden vibrational modes that are inactive in the BF-EELS. **c**, Comparison of signal delocalisation between BF- and DF- EELS of the dPS-*b*-P2VP lamellar structure. The CCD counts of the C–H signals on the P2VP domain are normalised, whereas the ADF-STEM intensity is min-max normalised. BF-EELS detects more than 50% of the C–H signal of the P2VP domain even on the dPS domains. **d**, Core-loss spectrum of the dPS-*b*-P2VP specimen acquired in the bright field shown in Fig. 2. Source data

Imaging techniques distinguishing between C–D and C–H were first applied to BCPs, polymers composed of two or more chemically distinct blocks linked to form a single chain. BCPs self-assemble into nanoscale periodic structures, known as microphase-separated structures, which vary in shape based on the ratio of the two polymer blocks^36^. Our initial study focused on the typical lamellar structure of a dPS and P2VP (dPS-*b*-P2VP) (Fig. 2a), where a carbon atom in the phenyl ring of PS is substituted with a nitrogen atom. Given the distinct chemical structures and immiscibility of dPS and P2VP, the microphase-separated lamellar structure was evident in the *Z*-contrast of the STEM-annular dark-field (ADF) images (Fig. 2b), with dPS and P2VP appearing as bright and dark contrasts, respectively. Estimated from the ADF profile, the domain spacings for dPS (*D*_dPS_) and P2VP (*D*_P2VP_) were approximately 31 nm and 37 nm, respectively (Extended Data Fig. 2). The domain size (*D* = *D*_dPS_ + *D*_dPS_) was ~68 nm, as per the STEM-ADF image, aligning well with findings from recent small-angle X-ray scattering experiments on symmetric PS-*b*-P2VP BCPs with similar molecular weights, which had a domain size (*D*) of ~60 nm (refs. ^37,38^).

**Extended Data Fig. 3:**
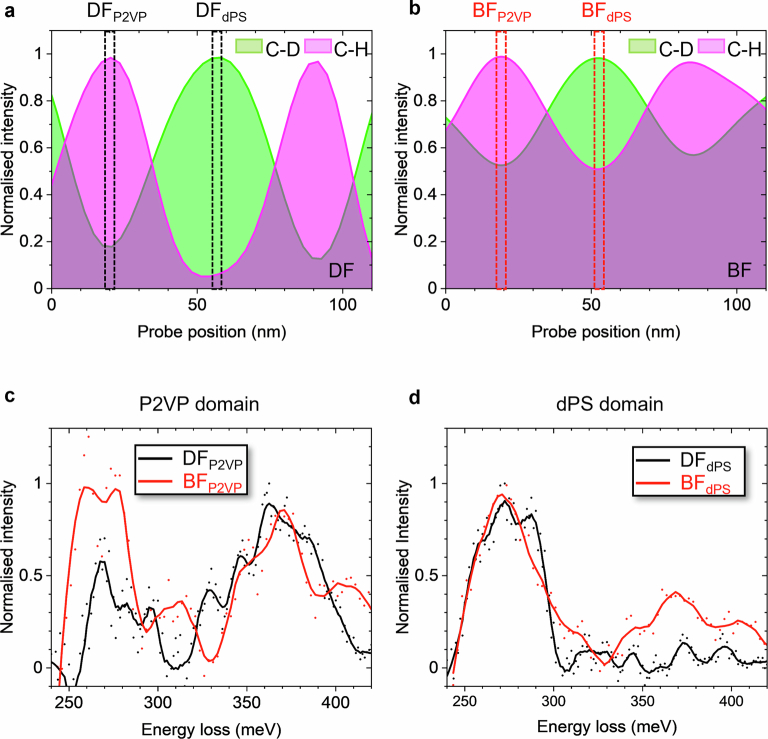
Comparison of signal delocalisation between DF-EELS and BF-EELS conditions. **a**, **b**, Intensity profiles of C−H (purple line) and C−D (green line) stretching peaks obtained by line scanning with BF- and DF-EELS perpendicular to the dPS-*b*-P2VP lamellar structure, respectively. The intensity profiles were processed to remove noise using an FFT filter and normalised to their respective maximum values. The purple and green peaks in **a** and **b** correspond to the centres of the P2VP and dPS domains, respectively. **c**, **d**, Comparison of the BF and DF spectra obtained in the P2VP and dPS domains. Each spectrum represents the integral of the area outlined by the dashed squares in **a** and **b**. Source data

A spectral image was collected from a 31 nm × 281 nm rectangular region (Fig. 2d) that contains four cycles of the lamellar structure. The spectrum acquired for this entire region is indicated in black in Fig. 2c, where the peaks at approximately 250–300 meV and 300–400 meV correspond to the C–D and C–H stretching vibrations, respectively. The intensity variations of these peaks, as well as for the N K-edge, are shown as colour maps in Fig. 2e–g and normalized intensity profiles in Fig. 2h. The spectra integrating regions I (P2VP), II (boundary) and III (dPS) in Fig. 2h are shown in purple, red and green, respectively, in Fig. 2c. Interestingly, the normalized intensity profiles show a notable extension of the C–D signal into the P2VP domain (Fig. 2h). In fact, the spectrum of the dPS domain showed only the C–D stretching peak (green line in Fig. 2c), whereas that of the P2VP domain revealed both the C–D and C–H stretching peaks, with the intensity of the former of these peaks at about 20% of that in the dPS domain (purple line in Fig. 2c). This signal extension cannot be attributed to signal delocalization, which is substantially smaller in DF-EELS compared with BF-EELS (Extended Data Fig. 2). Therefore, this is likely due to the segregation of dPS at the microtome section surfaces, as the surface free energy of dPS (*F*_surf,dPS_ = 29–37 mJ m^−^^2^) is lower than that of P2VP (*F*_surf,P2VP_ = 47–50 mJ m^−^^2^). The same approach was used to characterize the sample using BF-EELS (Extended Data Fig. 3). Although the peak intensity profile of BF-EELS corresponded with the lamellar structure, strong signals from the adjacent domains were detected owing to signal delocalization, which obscured local features, such as the surface segregation of dPS observed with DF-EELS.

## MD simulation of dPS-*b*-P2VP

**Fig. 3:**
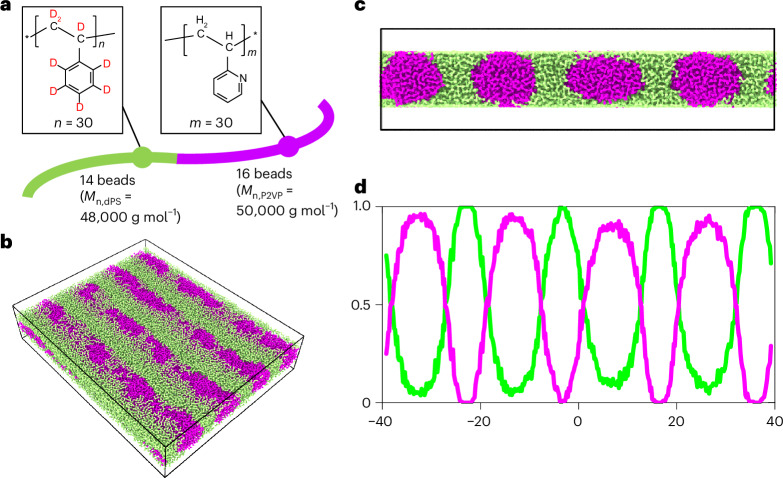
Microphase-separated structure of the dPS-*b*-P2VP BCP simulated with CGMD. **a**, Coarse-grained polymer chain of dPS-*b*-P2VP used for the CGMD simulation. Each coarse-grained bead contains 30 monomer units. The dPS and P2VP blocks consist of 14 and 16 beads, respectively. **b**, Entire structure of a snapshot of the equilibrated configuration of the dPS-*b*-P2VP system. **c**, Cross-sectional view of the structure shown in **b**. **d**, Composition profiles of dPS (green) and P2VP (purple) corresponding to **c**. Source data

To confirm this experimental finding, we performed CGMD calculations on dPS-*b*-P2VP by constructing flexible chains^39^ as a model to simulate the domain structures considering the difference in surface free energy (further details in Methods). The coarse-grained model used in this calculation is shown in Fig. 3a. The coarse-grained length unit (*σ*), that is, the length of a bead, can be transformed into a real unit of 4.2 nm to reproduce the sub-chains of BCPs at the same *χN* of ~115 at 120 °C (the Flory–Huggins interaction parameter *χ* and degree of polymerization *N* are 0.127 and 905, respectively)^40^. Our calculations clearly showed a wraparound structure, that is, the segregation of dPS at the surface (Fig. 3b,c). Consequently, we obtained the composition profiles of dPS-*b*-P2VP (Fig. 3d), wherein 20% of dPS was segregated in the P2VP domain, which is in good agreement with the experimental results. Notably, such surface segregation can neither be corroborated by conventional TEM imaging techniques nor by SANS.

## Spectral imaging of dPS-*b*-hPS

**Fig. 4:**
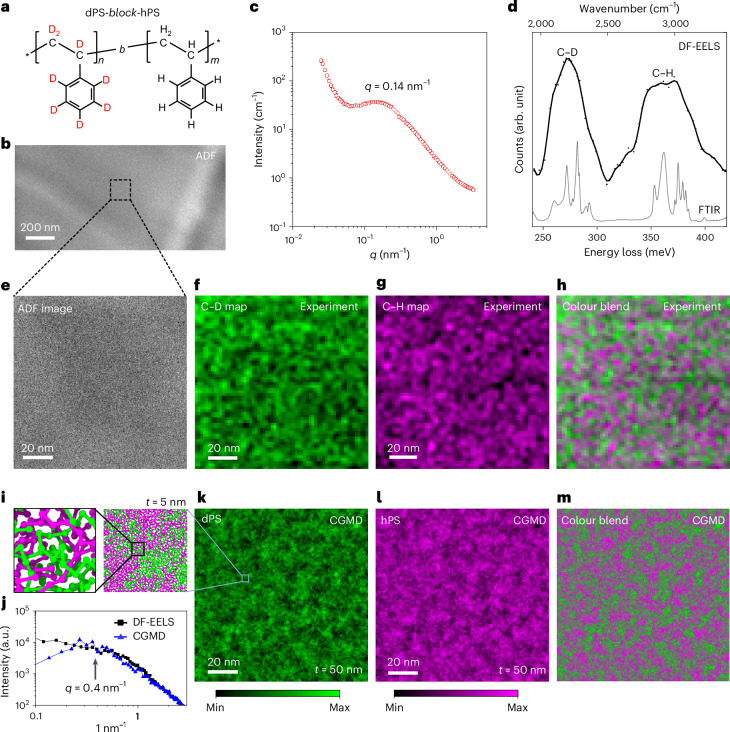
Hydrogen and deuterium mapping in the quenched melt of the dPS-*b*-hPS BCP. **a**, Chemical structure of dPS-*b*-hPS. **b**, ADF image of the microtome dPS-*b*-hPS thin film. **c**, One-dimensional SANS profile of the dPS-*b*-hPS specimen. The maximum scattering peak is at *q* = 0.14 nm^−1^, indicating that the characteristic domain size of dPS-*b*-hPS is approximately 45 nm in real space. **d**, Dark-field vibrational spectrum of dPS-*b*-hPS (black) compared with the FTIR spectrum. **e**, Two-dimensional-scanned area in **b** (158 nm × 158 nm). The area in **e** was scanned using a grid of 80 × 80 pixels (2.0 nm per pixel), with each parent pixel further subdivided into a 16 × 16 sub-scan. **f**–**h**, Colour maps of dPS (**f**), hPS (**g**) and their colour blend (**h**), derived from the C–D and C–H stretching peak intensities in **d**. The exposure time of a single pixel (2.0 nm × 2.0 nm) on the maps was 0.2 s. The colour maps were smoothed with a 3 × 3 low-pass filter. **i**, CG model of a quenched melt structure of the dPS-*b*-hPS BCP, from which a slice with a thickness (*t*) of 5 nm is extracted to show the polymer chains. **k**–**m**, Density profiles of dPS (**k**), hPS (**l**) and their colour blend (**m**) in a 50-nm-thick film simulated by CGMD, where the contrast is aligned to the min–max range. The simulated density maps of dPS and hPS, shown with colour according to the local thicknesses, are also presented in Extended Data Fig. 6. The spectral images were processed by the principal component analysis algorithm using five components. The C–D/C–H colour-mixed maps, along with the unfiltered maps, are presented in Extended Data Fig. 5. **j**, Comparison of the Fourier spectrum of the simulated density maps of dPS from **k** (*N* = 651) with the one obtained experimentally from **f**. Source data

Next, we investigated the concentration fluctuations in a thin specimen of a quenched melt of a di-block copolymer composed of chemically identical but isotopically different components, that is, dPS-*b*-hPS (Fig. 4a) with sufficiently small *χN*. The *χN* of the dPS-*b*-hPS was 0.41 at 120 °C (*χ* and *N* are 2.2 × 10^−4^ and 1,862, respectively), which provides Gaussian coil behaviour. The ideal radius of gyration for this BCP (*R*_g,0_) was approximately (*N*/6)^1/2^*b* ~12.5 nm, where *b* = 0.71 nm (ref. ^41^).

The one-dimensional SANS profile of the dPS-*b*-hPS specimen (Fig. 4c) exhibited a broad single peak with the peak maximum at *q* = 0.14 nm^−1^, corresponding to the value of *q* = 1.95/*R*_g,0_ predicted by random phase approximation^42^. Because SANS does not provide local structural information about the polymer chains except for the statistical *R*_g,0_, we applied DF-EELS as a real-space imaging technique to directly observe the molecular-level distribution of polymer chains.

Figure 4b,e presents the STEM-ADF images of the dPS-*b*-hPS specimen, where the contrast does not allow for the distinction of the respective components. The subtle contrast variations seen in Fig. 4b are attributed to thickness differences resulting from the ultramicrotomy cutting process. The spectrum obtained from this entire area, shown in Fig. 4d alongside the FTIR spectrum, clearly differentiates between the C–D and C–H stretching peaks. Intensity maps of these vibrational peaks, derived from the region with minimal thickness variation (Fig. 4e), are presented in Fig. 4f,g. These maps depict the distribution of dPS and hPS, with intensity variations potentially indicative of density fluctuations within each component. A colour-blended map combining both components is shown in Fig. 4h. A Fourier spectrum of the map (Fig. 4f) exhibited a shoulder around q^*^ = 0.4 nm^−1^, which is located on the lower *q* side of the peak detected by SANS (Fig. 4j). Note that the smallest discernible characteristic size in these maps, approximately 10 nm or less, corresponds to the region of *q* ≲ 0.6 nm^−1^ in reciprocal space.

## MD simulation of dPS-*b*-hPS

To elucidate the origin of the characteristic structure found in the experimental density maps, we performed a CGMD simulation of dPS-*b*-hPS, modelling it with semi-flexible chains^43^ (Fig. 4i), which accurately replicated the SANS profile. Figure 4k–m shows the CGMD-simulated density profiles for dPS, hPS and their colour blend within a quenched melt structure of dPS-*b*-hPS (*σ* ~0.76 nm) with a sample thickness of 50 nm, mirroring the dimensions of the experimental specimen. The intensity variations on these simulated density maps align with those observed on the experimental C–H and C–D maps.

**Extended Data Fig. 7:**
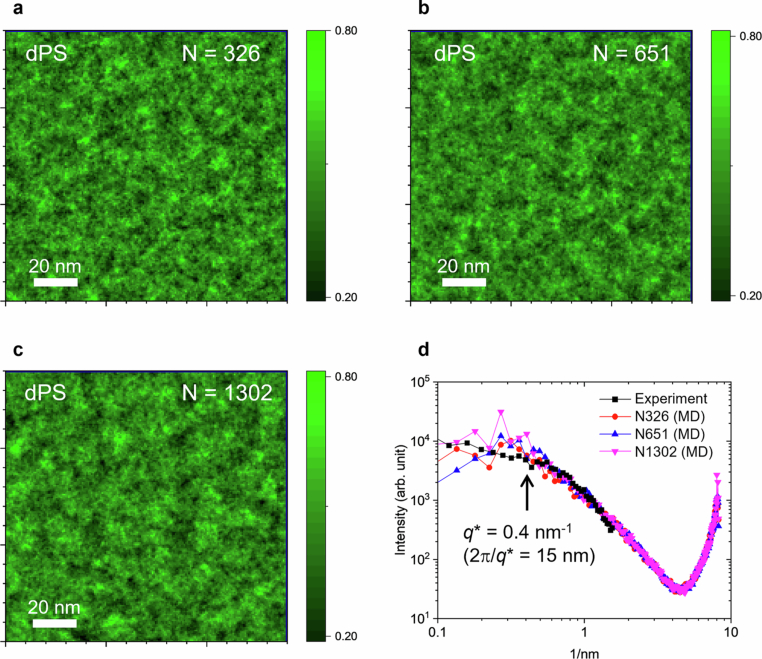
CGMD-simulated density distributions of dPS in a quenched melt of the dPS-*b*-hPS BCP for different chain length. **a**-**c**, Density profiles of dPS in a 50-nm-thick film simulated via the CGMD for *N* = 326 (half-length), *N* = 651 (full-length) and *N* = 1302 (double-length), respectively. **d**, Fourier spectrum of the simulated density profiles of dPS for each chain length as well as that of the experiment obtained from Fig. 4f. The peak top position (q^*^ = 0.4 nm^−l^) is almost consistent with all the calculated chain lengths and the experimental result. The size obtained from this peak (2π/q^*^ ~ 15 nm) corresponds to double the diameter of the reptation tube. Source data

In polymer melts, chains navigate within the tube-like topological constraint formed by the surrounding chains, known as ‘reptation tubes’. Reptation theory^32,33^ posits that the characteristic size of density fluctuations seen in simulated maps corresponds to the diameter of these reptation tubes (*d*_tube_). This is because adjacent chains situated within a distance less than *d*_tube_ demonstrate notable spatial correlations. For PS at 423 K, *d*_tube_ is approximately 7.6 nm (ref. ^43^). Notably, *d*_tube_ is almost independent from the chain length, that is, *R*_g,0_, as shown in Extended Data Fig. 7. When the thicknesses of specimens are less than several times of *d*_tube_, the density fluctuations caused by the reptation tubes should be observable. Indeed, a notable correlation exists between the Fourier spectrum of the simulated density map of dPS (Fig. 4k) and that of the experimentally obtained C–D map (Fig. 4f), as shown in Fig. 4j. The peak maximum in these spectra corresponds to approximately 15 nm, which indicates the period of the reptation tube (2*d*_tube_). This agreement suggests that the characteristic size in the intensity map for C–H and C–D stretches obtained from our experiments most likely originates from the density fluctuation of hPS and dPS caused by the reptation tubes. Such a structural feature has never been identified by SANS.

**Extended Data Fig. 8:**
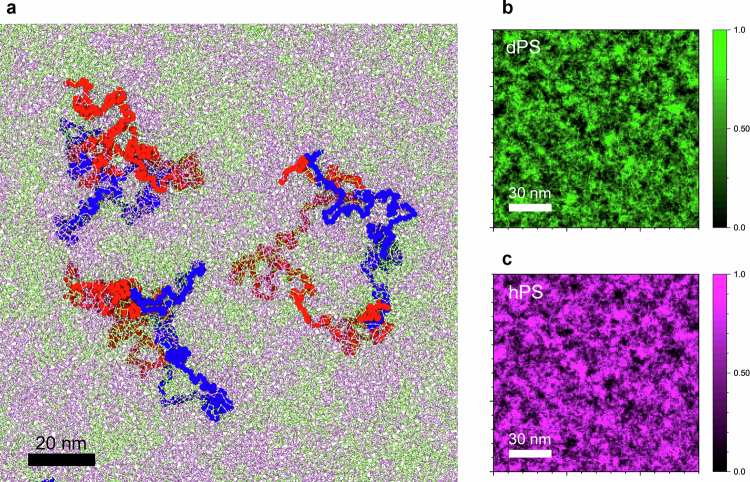
CGMD-simulated tube structures in a melt of the dPS-*b*-hPS BCP. **a**, Melt structure of the dPS-*b*-hPS BCP confined to a thin film (Thickness: 7.6 nm) (left panel). 450 dPS-*b*-hPS chains are packed in a region of approximately 185*σ* × 185*σ* × 10*σ*, maintaining a density of 0.85. The rough appearance of the reptation tubes of the polymer chains (shown in red and blue) are visualised. As presented in ref. ^39^, superimposition of the successive snapshots would be expected to more clearly show the reptation tubes. **b**, **c**, Density profiles of dPS and hPS in the 7.6-nm-thick film. Source data

This finding opens the possibility of further quantitative characterizations of individual polymer chains and reptation tubes. Thinner specimens (less than 10 nm) to prevent the overlapping of polymer chains along the thickness direction, as demonstrated in Extended Data Fig. 8, would provide direct visualization and more precise characterizations of polymer chains.

## Evaluation of electron beam damage

**Extended Data Fig. 9:**
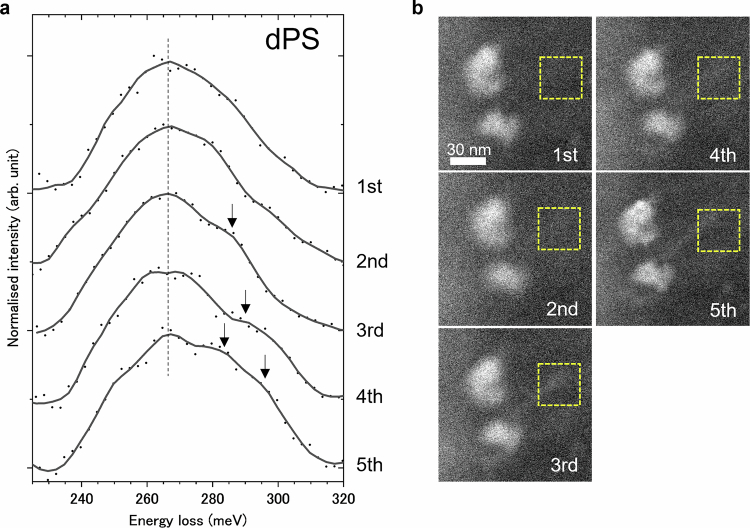
Investigation of electron beam damage to a dPS thin film. **a**, C−D stretching peaks obtained from 2D spectral images captured repeatedly at the same position. The measurement area is indicated by the yellow dashed lines in the ADF images shown in **b**. To ensure measurements were taken at the same position, we utilised the impurities (visible as white contrasts) as markers. The probe conditions matched those shown in Fig. 3, with a dose per frame of approximately 1×10^9^ e nm^−2^. The scanned area consisted of 15 × 15 pixels, with each pixel of dimensions 3.0 nm × 3.0 nm. Notably, there was an alignment time of ~10 min between two consecutive measurements. The spectra of the first and second measurements were largely similar. However, in the third measurement, slight changes were observed in the fine structure of the spectrum (evident from the sub-peaks indicated by black arrows). The ADF image revealed a slight accumulation of contaminants on the surface of the measurement area at this point. In the fourth and fifth measurements, the spectral width increased further. Source data

The electron beam damage incurred by the sample when using this method was evaluated by performing repeated two-dimensional (2D) scans to acquire vibrational spectra at the same position in a dPS thin film (Extended Data Fig. 9). We observed that the vibrational spectra remained nearly unchanged up to a cumulative electron dose of 2 × 10^9^ e nm^−2^, which is more than twice the electron dose used for the measurements in Figs. 2 and 4. This indicates that electron beam damage in those experiments was almost negligible. However, further exposure to the electron beam resulted in modifications to the fine structure of the vibrational spectra, suggestive of the radiolysis damage reported previously^44^.

**Extended Data Fig. 10:**
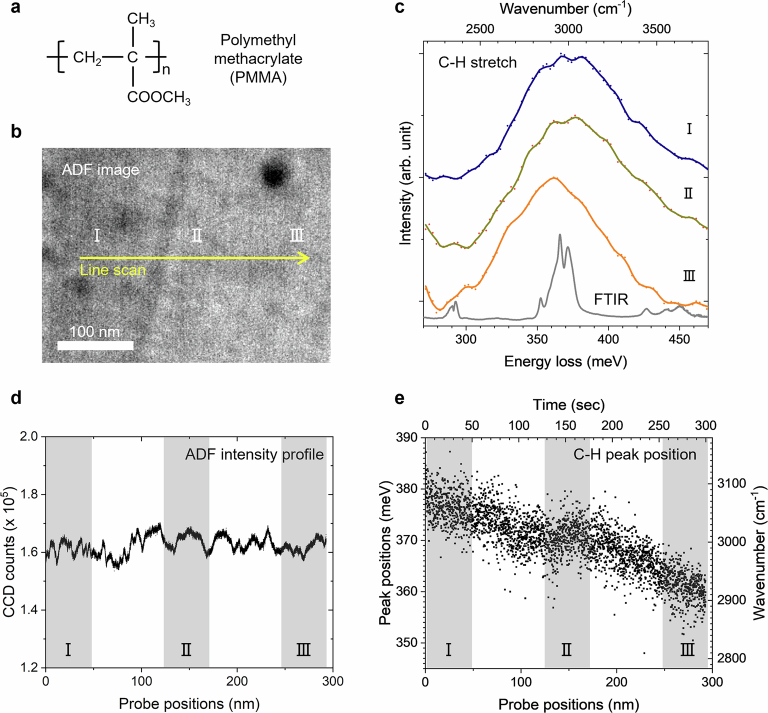
Variation in C−H stretching peak induced by electron beam damage to PMMA. **a**, Chemical structure of PMMA. **b**, STEM-ADF images of spin-coated PMMA thin films. **c**, Dark-field vibrational spectra of PMMA extracted from the 1D spectral image along the yellow line in (**b**). The FTIR spectrum of PMMA is in grey. **d**, ADF intensity profile during line scan. **e**, Variation in the C–H peak position during the line scan, with redshift of almost 20 meV. The peak positions in (**e**) are extracted from Gaussian fitting. Source data

We also examined the impact of electron beam damage on organic samples using poly(methyl methacrylate) (PMMA), known as one of the most beam-sensitive polymers (Extended Data Fig. 10). Vibrational spectra of the C–H stretches, acquired through DF-EELS from a spin-coated PMMA film, revealed slight yet noticeable alterations in the fine structure even within a line scan. By contrast, the concurrently obtained STEM-ADF intensity profile was nearly uniform. This result indicates that chemical bond alterations commenced at lower electron doses (less than 1 × 10^9^ e nm^−2^), which minimally reduced the thickness of the specimen.

## Conclusion

This study successfully visualized the nanoscale distribution of C–D and C–H bonds in polymers using DF-EELS to obtain C–D and C–H stretching vibrational spectra. The local structures observed in both phase-separated and melted BCPs align with predictions from CGMD simulations and reflect the inherent properties of the materials. Our approach provides access to molecular-scale structures in real space and offers advantages over traditional methods that rely on averaged data from reciprocal space. This nanoscale insight from real-space spectroscopy is anticipated to benefit polymer science and other disciplines involving organic materials, including bioscience.

## Methods

### Vibrational spectroscopy

All STEM-EELS experiments were conducted using TEM (JEOL Triple C#2) equipped with STEM and TEM DELTA-type spherical aberration correctors and a monochromator. The microscope operated at 60 kV with a probe current of approximately 13 pA. Nanoscale vibrational spectroscopy was carried out in STEM mode, where the convergence semi-angle of the incident beam and the EELS collection semi-angle in the dark field were set to 50 mrad and 20 mrad, respectively. The spatial resolution of the DF-EELS matches the probe size^33^, established at 2 Å based on the full-width at half-maximum and 2.7 Å at *D*_59_ (the diameter at which 59% of the electron current is contained within the probe). Electron energy loss (EEL) spectra were collected using a Gatan GIF spectrometer, tailored for low-voltage TEM, with the bright-field energy resolution of the electron probe at a full-width at half-maximum of 22 meV. The spectra, including the full zero-loss peaks, are shown in Extended Data Fig. 1. In addition, the polymer samples were examined at −120 °C using a MelBuild low-temperature holder. Background corrections for the DF-EEL spectra in Figs. 2 and 4 utilized a power-law function, with fitting windows positioned before and after the peaks of the C−D and C−H stretches, approximately at 230−240 meV and 420−430 meV, respectively. The spectral images used in Figs. 2 and 4 were processed using principal component analysis with a plug-in developed by HREM Research. The reconstructed images, utilizing five components, were used to generate the C−H/C−D maps. All these colour maps were generated from spectra with background subtraction. The pixel dimensions for the 2D scans shown in Figs. 2 and 4 are 3.1 nm × 3.1 nm and 2.0 nm × 2.0 nm, respectively. Each pixel was further subdivided into a 16 × 16 sub-scan, with each sub-pixel being nearly comparable to the probe size (~2 Å).

### Sample preparation

The dPS*-b*-P2VP and dPS*-b*-hPS BCPs were purchased from Polymer Source and used without further purification. The total number-average molecular weights (*M*_n_) were 98,000 g mol^−1^ (*M*_n,dPS_ = 48,000 g mol^−1^, *M*_n,P2VP_ = 50,000 g mol^−1^) and 200,000 g mol^−1^ (*M*_n,dPS_ = 90,000 g mol^−1^, *M*_n,hPS_ = 110,000 g mol^−1^) for dPS*-b*-P2VP and dPS*-b*-hPS, respectively. The volume fraction of the dPS block was calculated as 0.43 and 0.48 for dPS*-b*-P2VP and dPS*-b*-hPS, respectively (the densities of dPS, hPS and P2VP are 1.13 g cm^−^^3^, 1.05 g cm^−^^3^ and 1.09 g cm^−^^3^, respectively). The *M*_w_/*M*_n_ were 1.11 and 1.16 for dPS*-b*-P2VP and dPS*-b*-hPS, respectively. Chloroform (CHCl_3_) was purchased from Fujifilm Wako Pure Chemical Corporation. Bulk BCP specimens were prepared via solvent casting for 5 days using a 10 wt% CHCl_3_ solution. Thereafter, the resultant sample was thermally treated at 120 °C for 5 days under vacuum. The annealed samples were quickly quenched on a pre-cooled metal surface with liquid nitrogen to preserve their morphologies. Subsequent ultramicrotomy (Leica EM UCT) at 25 °C produced microsections ~50 nm thick, which were collected on copper grids covered with a lacey carbon film, without further staining.

### FTIR spectroscopy

FTIR measurements of dPS*-b*-P2VP and dPS*-b*-hPS BCPs were recorded on an FT/IR 6200 instrument (Jasco) with a resolution of 2.0 cm^−1^ at room temperature in air.

### SANS

SANS measurements were performed at the JRR-3 research reactor of the Japan Atomic Energy Agency in Tokai, Japan. Measurements were conducted with sample-to-detector lengths in the range of 1–12 m using neutrons with a wavelength of 7.0 Å with Δ*λ*/*λ* = 0.10 full-width at half-maximum, which covered the scattering wavenumber *q* range of 0.0025–0.3 Å^−1^. The scattered neutrons were detected using a 2D multiwired ^3^He detector. The necessary data corrections such as dark count (electronic noise), subtraction and cell-scattering subtraction were performed. Following these corrections, the datasets were normalized to an absolute scale using the incoherent scattering of a thin polyethylene plate as a standard sample. In addition, the 2D data were circularly averaged and incoherent scattering subtraction was performed. The high intensity in the region lower than the peak position in the SANS profile shown in Fig. 4c would be attributable to the scattering from the rough surfaces and voids present inside the sample for the SANS measurement, which obeys Porod’s law. However, no voids and defects were found in the area subject to the STEM and spectral measurements shown in Fig. 4.

### CGMD

We considered two types of CGMD simulation, those based on the flexible and semi-flexible Kremer–Grest (KG) model^39^, to observe the mesoscopic structures of the dPS-*b*-P2VP and dPS-*b*-hPS BCPs. Here we regarded conformationally symmetric BCPs with asymmetric compositions; the physical properties of dPS, P2VP and hPS were similar because of their structural similarities^45^. We performed CGMD simulations on an AB-type di-block copolymer (di-BCP) chain with interaction parameters corresponding to the *χN* values of the specimens used for the experiments.

The flexible KG model incorporates the Lennard-Jones (LJ) potential for interactions between nonbonded KG beads and the finite extensible nonlinear elastic (FENE) bond potential for consecutive KG beads along the polymer chain. The semi-flexible KG model extends this by including bending-angle potentials, in addition to the LJ and FENE potentials present in the flexible model. We adhered to the standard parameters for the KG model in both cases. For ease of simulation, we set *σ* = *ε* = *τ* = *k*_B_ = 1 and *m* = 1, ensuring uniformity across mass segments, where *σ*, *ε* and *τ* represent the units of bead length, energy and time, respectively. All the simulations were performed at *T* = 1 and *P* = 0. Moreover, all simulations were performed using LAMMPS^46^. Visualizations of the molecular structures were generated using OVITO^47^, which provided clear snapshots for analysis.

### CGMD for dPS-*b*-P2VP

For the dPS-*b*-P2VP, the values of *χN* were reproduced in the simulation model. As the structure of the melted BCP at 120 °C was frozen at –120 °C during the experiments, the *χ* parameter (0.127) of the dPS-*b*-P2VP at *T* = 120 °C was applied. Because the degree of polymerization *N* of the examined dPS-*b*-P2VP (*M*_n,dPS_ = 48,000 g mol^−1^ and *M*_n,P2VP_ = 50,000 g mol^−1^) was approximately 905, *χN* was approximately 115.

To reproduce the structure di-BCPs form with a certain *χN*, we used the cut-off length *r*_c_ = 2.5*σ* for the LJ potential and adjusted the LJ interaction strength *ε*_AB_ between the A- and B-KG beads based on the mapping relation for the KG model: *χN* = *C* (1 − *ε*_AB_)*N*_KG_. On the basis of the relationship between *χ* and the interfacial width^36^, the estimated *χN* for *ε*_AB_ = 0.3 and *N*_KG_ = 30 was approximately 115. The numbers (*N*_KG,A_ and *N*_KG,B_) of the A-KG and B-KG beads were determined based on the volume fraction obtained from the molecular weight and bulk density of each component. Consequently, we set (*ε*_AB_, *N*_KG,A_, *N*_KG,B_) as (0.3, 14, 16).

To replicate the diffusion behaviour of the A (dPS) segments on the free surface, we sandwiched melts of the AB-type di-BCPs between parallel flat walls. This set-up was used because the structures and composition profiles observed are transient (quasi-stable) and influenced by surface free energy. To accurately simulate this quasi-stable state as a pseudo-equilibrium condition, CGMD simulations were used to fine-tune the interactions at the walls. Attractive forces were modelled between the walls and A-beads (dPS segments), as well as among the A-beads themselves, to mimic their affinity. Conversely, segregating interactions were established between the walls and B-beads, and between the A- and B-beads, to represent their mutual repulsion. The flat walls in the simulation consisted of thermostat and rigid-body beads, with the thermostat beads positioned at the polymer matrix interface to regulate the temperature. Among the A-, B- and thermostat (C-) beads, *ε*_AC_ = 1.03 and *ε*_BC_ = *ε*_AB_ were applied. The value of *ε*_AC_ was selected to reproduce the observed structures and composition profiles.

Periodic boundary conditions (PBCs) were implemented in the *x*- and *y*-axes, with the *z*-axis perpendicular to the parallel walls. We positioned 2,304 polymer chains within this set-up, adhering to PBCs and wall constraints, to emulate a system with a *D*:*W* of 19.6:13.1, where *D* represents the lamellar domain spacing and *W* the distance between the walls. The wall positions were determined to maintain the pressure *P*_*z*_ = 0 in the *z*-direction. We performed a CGMD run with 2 × 10^6^ MD steps and Δ*t* = 0.005 *τ*. The size of the polymer region became 98.4*σ* × 78.3*σ* × 13.1*σ* for *ε*_AB_ = 0.3 and *ε*_AC_ = 1.03.

Notably, the length of the PBCs in the *y*-direction, perpendicular to the lamellar structure, was shortened by nearly 5% compared with the bulk case. This adjustment was made with the expectation that the width of the P2VP domains (*D*_P2VP_) under confinement would remain consistent with their bulk counterpart, while the width of the dPS domains (*D*_dPS_) would decrease owing to the diffusion of molecules towards the free surfaces.

### CGMD for dPS-*b*-hPS

For the dPS-*b*-hPS, we used a semi-flexible chain model to reproduce the rheological properties^43^ because *χN* is approximately 0. On the basis of the mapping^43^ between the semi-flexible chain model and actual PS chain, we set (*ε*_AB_, *N*_sKG,A_, *N*_sKG,B_, *κ*) as (1.0, 281, 370, 0.944), where *κ* is the strength of the bending-angle potential. In this mapping^43^, *σ* corresponds to the length of a KG bead of 0.76 nm.

To estimate the structural factors within a wide *q* range, 8,100 chains were arranged in a PBC box replicated in the *x*-, *y*- and *z*-directions. The dimensions of this PBC box were set to 183.7*σ* in each direction. The cut-off length for the LJ potential was designated as *r*_c_ = 2^1/6^*σ*, with a number density (*ρ*) of 0.85. To map the density distributions of the dPS and hPS segments, we determined the equilibrium positions of the homopolymer melts (with a Flory–Huggins interaction parameter, *χN* ~0) by using an advanced version^48^ of the double-bridge hybrid method^49^. The peak position (q^*^ ~0.2*σ*^−1^) of the structure factor *S* (*q*) of the dPS components of the obtained polymer configuration supported the SANS experiment shown in Fig. 4c.

## Online content

Any methods, additional references, Nature Portfolio reporting summaries, source data, extended data, supplementary information, acknowledgements, peer review information; details of author contributions and competing interests; and statements of data and code availability are available at 10.1038/s41565-025-01893-5.

## Source data

Source Data Fig. 1 EELS and FTIR spectral data of dPS.

Source Data Fig. 2 EELS spectral data of dPS-*b*-P2VP and EELS intensity profiles.

Source Data Fig. 3 Computed compositional profile data of dPS and P2VP.

Source Data Fig. 4 SANS data, EELS and FTIR spectral data of dPS-*b*-hPS, EELS intensity profiles for each colour map, and Fourier spectral data of density maps.

Source Data Extended Data Fig./Table 1 EELS spectral data of PS.

Source Data Extended Data Fig./Table 2 EELS spectral data of dPS for DF and BF conditions, intensity profile data of DF, BF and ADF, and core-loss spectral data of dPS-*b*-P2VP.

Source Data Extended Data Fig./Table 3 EELS intensity profile data of dPS-*b*-P2VP for DF and BF and EELS spectral data of dPS-*b*-P2VP.

Source Data Extended Data Fig./Table 4 EELS spectral data of dPS-*b*-P2VP for a single pixel.

Source Data Extended Data Fig./Table 5 EELS intensity profile data for each colour map before and after filtering.

Source Data Extended Data Fig./Table 6 Computed density profiles for each colour map.

Source Data Extended Data Fig./Table 7 Computed compositional profile data of dPS and hPS and Fourier spectral data of colour maps.

Source Data Extended Data Fig./Table 8 Computed compositional profile data of dPS and hPS.

Source Data Extended Data Fig./Table 9 EELS spectral data of dPS.

Source Data Extended Data Fig./Table 10 EELS spectral data of PMMA, ADF intensity profile data and C–H peak position’s plots during a line scan.

## Figures and Tables

**Extended Data Fig. 4:**
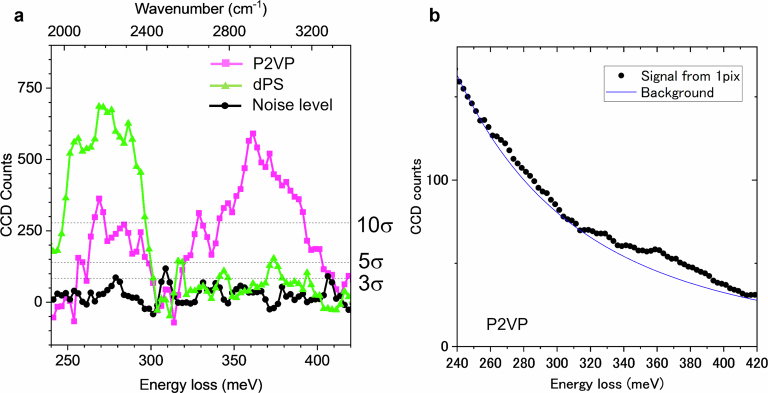
Spectral reliability at the single-pixel level. **a**, Comparison of the signals from the C−D and C−H stretching peaks with the noise levels (expressed as the standard deviation, σ, of the noise signal) obtained from a single pixel in the P2VP and dPS domains shown in Fig. 2. The noise level was determined using the spectrum of carbon film, which lacks C−H and C−D peaks, under the same electron beam conditions. **b**, Raw data (black points) and the corresponding background fit (blue line) from a spectrum obtained at a pixel within the P2VP domain. A power-law function was used to model the background. The vertical unit in **a** and **b** is expressed in charge coupled device (CCD) counts. Source data

**Extended Data Fig. 5:**
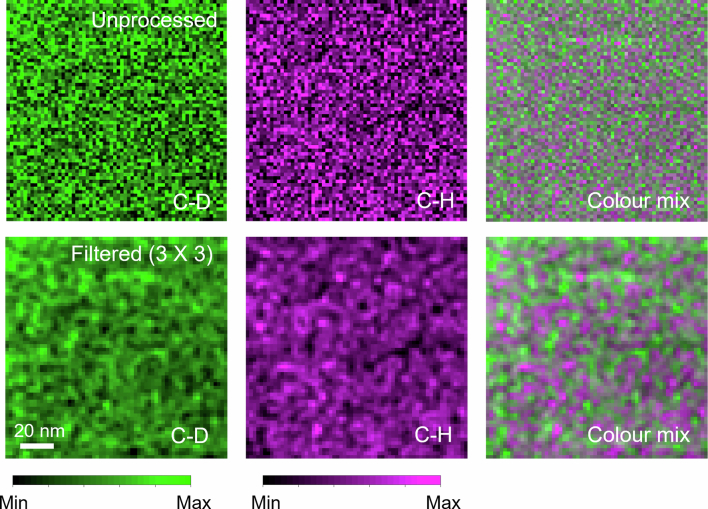
C−D and C−H stretching peak intensity maps for dPS-*b*-hPS. The top panels display the maps before filtering: C−D (left), C−H (middle), and a colour blend (right). The bottom panels show the corresponding maps after filtering, which were used in Fig. 3. Source data

**Extended Data Fig. 6:**
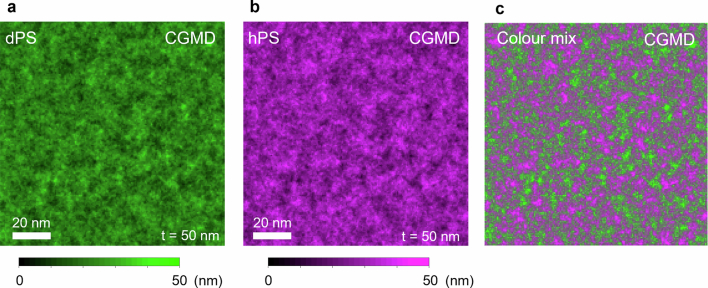
CGMD-simulated density distributions of dPS and hPS in a quenched melt of the dPS-*b*-hPS BCP. **a**,**b**, Density profiles of dPS and hPS in a 50-nm-thick film simulated via the CGMD, where their contrasts indicate the thicknesses of the dPS and hPS components, respectively. **c**, Colour-mixed map of **a** and **b**. Source data

## Data Availability

The datasets generated and/or analysed in this study are provided with the paper as source data. Source data are provided with this paper.

## Code availability

LAMMPS is an open-source classical molecular dynamics code distributed at https://www.lammps.org.
