# Author Correction: Nanoscale C–H/C–D mapping of organic materials using electron spectroscopy

**DOI:** 10.1038/s41565-025-02040-w

**Published:** 2025-10-17

**Authors:** Ryosuke Senga, Katsumi Hagita, Tomohiro Miyata, Hsiao-Fang Wang, Koichi Mayumi, Hiroshi Jinnai, Kazu Suenaga

**Affiliations:** 1 Nanomaterials Research Institute, National Institute of Advanced Industrial Science and Technology (AIST), Tsukuba, Japan; 2 SANKEN (The Institute of Scientific and Industrial Research), The University of Osaka, Ibaraki, Japan; 3 Department of Applied Physics, National Defense Academy, Yokosuka, Japan; 4 Institute of Multidisciplinary Research for Advanced Materials, Tohoku University, Sendai, Japan; 5 Department of Chemical and Materials Engineering, National Central University, Taoyuan City, Taiwan; 6 The Institute for Solid State Physics, The University of Tokyo, Kashiwa, Japan

**Keywords:** Polymer characterization, Transmission electron microscopy, Characterization and analytical techniques, Molecular dynamics

Correction to: *Nature Nanotechnology* 10.1038/s41565-025-01893-5, published online 24 March 2025.

This article was originally published under standard Springer Nature license (© The Author(s), under exclusive licence to Springer Nature Limited). It is now available as an open-access paper under a Creative Commons Attribution 4.0 International license, © The Author(s). The error has been corrected in the HTML and PDF versions of the article.

